# Content of Minerals and Fatty Acids and Their Correlation with Phytochemical Compounds and Antioxidant Activity of Leguminous Seeds

**DOI:** 10.1007/s12011-017-1005-3

**Published:** 2017-03-29

**Authors:** Eugeniusz R. Grela, Wioletta Samolińska, Bożena Kiczorowska, Renata Klebaniuk, Piotr Kiczorowski

**Affiliations:** 1 Institute of Animal Nutrition and Bromatology, University of Life Sciences, Akademicka 13, 20-934 Lublin, Poland; 2 Department of Biological Bases of Food and Feed Technologies, University of Life Science, Głęboka 28, 20-612 Lublin, Poland

**Keywords:** Leguminous seeds, Elements, Fat profile, Nutritional value, Antioxidant activities, Antinutritional

## Abstract

The aim of the study was to determine the mineral composition and fatty acid profile in the seeds of selected Fabaceae species and cultivars and to assess their correlations with phytochemicals and antioxidant activity. The Andean lupine was characterised by a particularly high level of Mg and K as well as Cu, Zn, and Fe (*P* < 0.05). There were various correlations (*P* < 0.05) between the total phenols and tannins and these elements. The highest contribution of α-linolenic acid (ALA, 18:3, *n*-3) in total fatty acids was noted in the lentil (13.8 in 100 g^−1^ fat), common bean (11.9 in 100 g^−1^ fat), and pea seeds (10.4 in 100 g^−1^ fat) (*P* = 0.028). In turn, the white lupine contained the highest content of ALA—0.67 g 100 g^−1^ seeds; its lowest level was determined in the broad bean—0.03 g 100 g^−1^ seeds. The seeds exhibited a high proportion of hypocholesterolemic fatty acids (on average 86%). The 2,2-diphenyl-1-picrylhydrazyl antiradical activity was positively correlated with UFA and PUFA (*P* < 0.05). This indicates great protective potential of legume seeds for prevention and treatment of diet-dependent diseases.

## Introduction

The UNO General Assembly has declared the year 2016 as the International Year of Pulses (IYP) [1]. IYP 2016 is designed to raise public awareness of the nutritional value of legume plants in order to improve food and nutrition security. Legume seeds are a valuable element of a healthy diet due to their content of protein with a high biological value. Additionally, they are a source of other valuable nutrients [2, 3]. For instance, they contain minerals such as magnesium, calcium, and iron, which are usually insufficiently provided in the human diet [4], and unsaturated fatty acids with their protective effect in prevention and treatment of cardiovascular diseases [2, 3, 5]. Furthermore, legume seeds contain a number of bioactive phytochemicals (flavonoids, phenolic acids, carotenoids, phytosterols, and phytohaemagglutinins) with a wide spectrum of activity. These compounds often exhibit complementary and overlapping mechanisms of action, including stimulation of the function of the immune system, modulation of lipid metabolism, inhibition of lipoprotein oxidation and platelet aggregation, as well as anticarcinogenic, antiangiogenic, and antimutagenic properties [2, 3]. Although protease inhibitors, lectins, alkaloids, and phenol derivatives, i.e. tannins, are traditionally regarded as antinutritional compounds, a number of studies have revealed their health-enhancing effect. There are reports of their efficiency in the prophylaxis and treatment of obesity, diabetes, and hypertension as well as their antimutagenic and antimicrobial effects [2, 6, 7]. The biological activity of non-nutritive phytochemicals is often associated with their antioxidant capacity. Many of these compounds are exogenous for the human organism; therefore, supply thereof with food has a preventive effect, particularly in protection against free radicals. Plant phenolic compounds with antioxidant activity include flavonoids, tannins, phenolic acids, stilbenoids, and lignans, which are present in legume seeds [2, 8–10].

Currently, there is little information concerning the interrelationships between minerals and phytochemicals contained in legume seeds [11] and between groups of fatty acids and antioxidant activity [12].

Therefore, the aim of the study was to determine the mineral composition and fatty acid profile in the seeds of selected Fabaceae species and cultivars and to assess their correlations with phytochemicals and antioxidant activity determined in our previous studies [13].

## Materials and Methods

### Seed Sample

Approximately 5 kg of the legume seeds studied (Table 1) was obtained from cultivation areas of South and East Poland, while *Cicer arietinum* and *Lens culinaris* were obtained from Turkey. All the seeds were sown (5th April) in experimental plots (1.5 × 3 m, no edge rows) with 20-cm spacing in rows, 40 cm apart, at the Institute of Plant Genetics in Cerekwica, Poland (51° 55′ N, 17° 21′ E), in 2012 and 2013. Immediately after sowing, the Afalon herbicide was applied on the plots. No mineral fertilisation was applied during the growth and development of the plants.

**Table 1:** Origin and estimation of morphological traits of some legume species

Species	Country of origin	Plant height (cm)	Days to the first flowering	Flower colour	Number of pods per plant	Pod length (cm)	Number of seeds per pod	Seed coat colour	TSW (g)
*Lupinus angustifolius*									
cv. Regent	Poland	63.0	64	Blue	18	3.8	4.2	Grey with pigmentation	134.2
cv. Zeus	Poland	65.0	57	White to light pink	17	3.72	4.5	White	148.5
*Lupinus albus*									
cv. Butan	Poland	68.0	63	White/grey to slightly blue	17	4.0	3.4	Bright to cream	237.4
cv. Bardo	Poland	67.3	60	White to slightly blue	15.8	4.3	4.1	Cream	308.7
*Lupinus luteus*									
cv. Parys	Poland	85.2	68	Yellow to slightly orange	17.9	3.4	4.1	Bright with dark pigmentation	118.7
cv. Iryd	Poland	81.7	67	Orange	17.3	3.8	3.9	Grey	128.6
*Lupinus mutabilis* Sweet	Poland	95.4	69	Bright blue	55.1	4.1	5.9	Bright	82.2
*Pisum sativum*									
cv. Medal	Poland	62.5	58	White	20.5	3.8	4.2	Cream to bright yellow	249.7
cv. Mentor	Poland	87.8	62	White	23.4	4.1	4.4	Yellow	223.2
*Cicer arietinum*	Turkey	38.2	56	Light pink	41.8	1.1	1.9	Bright to cream	267.9
*Lens culinaris*	Turkey	37.6	55	White to slightly blue	58.0	1.9	1.8	Slightly brown to grey	33.7
*Lathyrus sativus*									
cv. Derek	Poland	43.0	57	White	37.8	3.2	2.9	Bright to cream	115.4
cv. Krab	Poland	43.5	60	Blue		3.9	3.3	Cream-greenish	147.3
*Phaseolus vulgaris* L.									
cv. Mela	Poland	23.0	37	White	8.4	12.21	3.1	White slightly veined	498.7
*Vicia faba* L.									
cv. Bonus	Poland	105.0	63	White	9.13	11.58	3.7	White to bright yellow or bright green	613.4

Throughout the vegetation period, morphological description of plants from the particular accessions was carried out. The plant growth habit was recorded at the onset of the flowering period and the flowering time as the number of days from sowing to the first flower opening. The flower colour was assessed on the dorsal petal in opened flowers. The plant height was measured at physiological maturity from the ground to the top of the longest shoot. After the harvest, the number of pods per plant, pod length, and number of seeds per pod were determined. The number of pods per plant was evaluated from five randomly selected plants per plot. The pod length and number of seeds per pod were constant in mature pods (10 per plant). Mature seeds were used for estimation of the seed coat colour. All the characteristics mentioned above were scored according to the suggestions specified in the IPGRI (International Plant Genetic Resources Institute) descriptors. This ensured uniform growth conditions for all plants, which produced the seeds collected for analysis. Three hundred to four hundred fifty grams portions of the original samples was hand sorted to remove splits, small wrinkled beans, and foreign materials. Thousand-seed weight (TSW) was determined as well (4 × 1000 seeds for repetition). A random sample was taken after harvesting for analysis of the content of minerals and fatty acids.

### Mineral Analysis

The chemical analysis involved determination of the content of Na^+^, K^+^, Ca^+2^, Mg^+2^, Zn^+2^, Cu^+2^, Fe^+2^, and Mn^+2^ in mineralised legume seed samples (*n* = 3). The contents of the elements were determined in the seed materials (2 g of legume seeds) after incineration in a muffle furnace at 480 °C. The resultant ash was solubilised on crucibles using 6 mol l^−1^ of spectrally pure hydrochloric acid (POCH, Poland). Na and K were analysed using flame atomic emission spectroscopy (FAES) with a flame photometer (Pye Unicam SP 2900, Cambridge, UK) at a wavelength of *λ* = 589.0 nm and *λ* = 766.5 nm, respectively. Ca, Mg, Zn, Cu, Fe, and Mn concentrations were determined using flame atomic absorption spectroscopy (FAAS) with a SOLAAR 939/959 spectrophotometer (Unicam, Cambridge, UK). Calcium was determined at *λ* = 422.7 nm, magnesium at *λ* = 285.2 nm, zinc at *λ* = 213.9 nm, copper at *λ* = 324.8 nm, iron at *λ* = 248.3 nm, and manganese at *λ* = 279.5 nm, according to the Polish Norm PN-EN ISO 6869:2002 [14]. The accuracy of the analytical procedure was verified by an analysis of certified reference materials for Multielement Trace Analysis Soya Bean Flour (INCT-SBF-4), manufactured by the Institute of Nuclear Chemistry and Technology (Warsaw, Poland). The recovery levels (*n* = 3) and relative standard deviations (RSD) for the analysed elements were as follows: Na (101.1%, 5.3%); K (97.6%, 6.4%); Ca (98.8%, 7.9%); Mg (99.5%, 5.1%); Zn (99.2%, 6.2%); Cu (96.1%, 5.8%); Fe (97.3%, 7.4%), and Mn (98.6%, 7.7%). The phosphorus content was determined with the spectrometric method at 400 nm using a Helios Alpha UV-VIS apparatus (Spectronic Unicam, Leeds, UK), according to AOAC [15].

### Fatty Acid Analysis

The fatty acid composition was determined with the gas chromatography method on a Varian CP-3800 chromatograph CP-3800 (Varian Inc., Palo Alto, USA) after conversion of the fats to fatty acids methyl esters (FAME) according to the AOAC method [16]. The chromatograph operating conditions for fatty acid separation were as follows: capillary column CP WAX 52CB DF 0.25 mm of 60 m length, gas carrier—helium, flow rate—1.4 ml min^−1^, column temperature 120 °C gradually increasing by 20 °C min^−1^, determination time—127 min, feeder temperature—160 °C, detector temperature—160 °C, and other gases—hydrogen and oxygen. The determinations were based on a template such as Supelco 37-Component Fame Mix (Sigma-Aldrich Poznań, Poland). The results were expressed as the proportion of individual fatty acids in the total value of fatty acids taken as 100%. Additionally, the content of desirable acids with a neutral effect on the cholesterol level as well as hypocholesterolemic acids (DFA) and the content of undesirable hypercholesterolemic fatty acids (OFA) were calculated [17]. The atherogenicity index (AI) was calculated from the fatty acid profile according to Ulbricht and Southgate [18].

### Statistical Analysis

The analyses were performed in triplicate and all data were expressed as means. The percentage data of fatty acids were arcsine transformed. The normality of data and homogeneity of variances were tested using the Shapiro-Wilk and Brown-Forsythe tests, respectively. The data obtained were analysed statistically using the species as an independent variable in the general linear model (GLM) of one-way ANOVA analysis of variance or non-parametric Kruskal-Wallis test (a non-parametric equivalent of one-way analysis of variance). Detailed comparisons between the means of legume species were conducted using the post hoc Duncan or Dunn test. Pearson’s or Spearman’s correlation tests were conducted to determine the linear correlations among some variables of the legume seeds. The following scale was used in the interpretation of the correlation coefficient: 0 < *r* < 0.3 a low degree of correlation; 0.3 ≤ *r* < 0.5 a moderate correlation; and 0.5 ≤ *r* < 1 a high degree of correlation. All statements of significance were based on the 0.05 probability levels. All the data of the chemical composition of the leguminous plant seeds were analysed with the Statistica software version 10.0.

## Results and Discussion

### Macro- and Microelement Content in Legume Seeds

Legume seeds can be an important source of such elements as manganese, copper, phosphorus, zinc, magnesium, iron, and even calcium and potassium in human nutrition [4]. In the present study, the content of macro- and microelements, except for sodium, was specific to the legume species (Table 2). All the leguminous plant seeds were characterised by high potassium (9–12 g kg^−1^ DM) and low sodium content (0.1–0.2 g kg^−1^ DM). The highest level of potassium was detected in the broad bean and white lupine (11.8 and 11.0 g kg^−1^ DM), and the lowest content of this element was determined in the grass pea, yellow lupine, and common bean seeds (below 9 g kg^−1^ DM) (*P* < 0.05). The phosphorus and calcium levels were dependent both on the species and on the variety (Table 2). The greatest amount of phosphorus in 1 kg (DM basis) was found in the yellow lupine (7.0 g) and broad bean (6.2 g), whereas its lowest quantity (3.0 g) was detected in the chickpea seeds (*P* = 0.008). The highest content of calcium (6.0–5.5 g) was noted in the chickpea and lentil seeds and the smallest quantity was noted (1.0–2.8 g kg^−1^ DM) in the seeds of the Andean lupine, broad bean, grass pea, common bean, and pea (*P* < 0.05). Similarly, the content of microminerals in the evaluated leguminous plant seeds was dependent on both the species and the variety, and the greatest differences were noted for manganese. Noteworthy, the white lupine seeds accumulated significant amounts of manganese [19]. The greatest levels of this element were noted in the white lupine seeds (especially in the Butan cultivar—370 mg kg^−1^ DM), compared with only 13–20 mg kg^−1^ DM in the lentil, grass pea, common bean, and broad bean seeds (*P* < 0.05) (Table 2). In these investigations, the average content of manganese was 252 g kg^−1^ DM, while the adequate intake (AI) of this element is 1.8 mg day^−1^ [20]. However, manganese, which is primarily supplied by vegetable food and water in the daily diet, is characterised by a very low level of absorption in the gastrointestinal tract (1–4%) [21].

**Table 2:** Mineral content of some leguminous species

Species		Macroelements, g kg^−1^ DM					Microelements, mg kg^−1^ DM			
		K	Na	P	Ca	Mg	Mn	Fe	Zn	Cu
Blue lupine	cv. Regent	9.31	0.16	4.62	2.72	1.39	26.49	35.09	34.43	4.64
	cv. Zeus	8.98	0.18	4.57	2.84	1.27	25.14	36.54	33.45	3.89
	Mean value	9.15^ab^	0.17	4.60^ab^	2.78^ab^	1.33^ab^	25.82^ab^	35.82^b^	33.94^bc^	4.27^c^
White lupine	cv. Butan	10.54	0.18	5.47	2.58	1.32	369.22	38.50	44.31	8.44
	cv. Bardo	11.45	0.15	4.98	2.18	1.37	135.45	39.41	42.56	7.89
	Mean value	11.00^a^	0.17	5.23^ab^	2.38^ab^	1.35^ab^	252.34^a^	38.96^b^	43.44^b^	8.17^a^
Yellow lupine	cv. Parys	8.55	0.16	7.18	1.76	2.22	59.48	54.85	55.85	8.92
	cv. Iryd	8.98	0.14	6.78	1.66	2.09	61.45	49.87	56.78	9.14
	Mean value	8.77^b^	0.15	6.98^a^	1.71^ab^	2.16^a^	60.47^ab^	52.36^a^	56.32^a^	9.03^a^
Andean lupine		11.69^a^	0.15	5.79^ab^	1.28^b^	2.17^a^	49.11^ab^	56.63^a^	52.71^a^	7.21^a^
Pea	cv. Medal	10.07	0.11	3.57	0.52	1.17	27.66	51.26	40.94	6.32
	cv. Mentor	11.45	0.09	4.12	0.49	1.14	24.45	49.58	38.47	5.48
	Mean value	10.76^ab^	0.10	3.85^b^	0.51^b^	1.16^ab^	26.06^ab^	50.42^ab^	39.71^b^	5.90^bc^
Chickpea		10.18^ab^	0.13	2.96^b^	6.04^a^	0.86^b^	19.21^ab^	39.08^ab^	27.49^b^	5.96^bc^
Lentil		9.66^ab^	0.14	3.98^b^	5.50^a^	1.71^ab^	12.66^ab^	62.94^a^	34.63^bc^	6.66^ab^
Grass pea	cv. Derek	8.71	0.15	4.68	0.97	1.14	13.29	43.52	29.57	6.98
	cv. Krab	9.23	0.19	5.13	1.03	1.24	15.31	42.45	31.24	7.25
	Mean value	8.97^b^	0.17	4.91^ab^	1.00^b^	1.19^ab^	14.30^ab^	42.99^ab^	30.41^c^	7.12^ab^
Common bean	cv. Mela	8.47^b^	0.16	4.79^ab^	0.98^b^	1.47^ab^	16.47^ab^	40.58^ab^	27.58^c^	8.12^a^
Broad bean	cv. Bonus	11.80^a^	0.20	6.15^a^	1.19^b^	1.26^ab^	17.74^ab^	43.79^ab^	48.13^ab^	10.14^a^
Pooled SEM		0.308	0.008	0.298	0.130	0.106	23.684	2.116	2.571	0.443
*P* value		0.030	0.118	0.008	0.048	0.027	0.036	0.008	0.007	0.001

Values are mean of triplicate analyses

^abc^Statistical differences between mean values (*P* < 0.05)

The greatest amounts of iron were noted in the lentil, Andean lupine, and yellow lupine seeds and its lowest content was detected in the seeds of the white and blue lupines (*P* = 0.008). Among the analysed legume seeds, the yellow and Andean lupines contained the highest levels of zinc, while the lowest content of this element was noted in the grass pea, common bean, and chickpea seeds (*P* = 0.007). The concentration of copper ranged from 4 to 10 mg kg^−1^ DM, with the lowest values noted in the white lupine (4 mg) and the highest in the seeds of the broad bean, yellow and white lupines, and common bean seeds (10–7 mg) (*P* = 0.001).

Cabrera et al. [22] and Özcan [23] reported that the levels of macroelements in Fabaceae were present in the following ranges: 7426–16,558 mg K kg^−1^; 269.75–445.81 mg Na kg^−1^; 2719–5556 mg P kg^−1^; 1309–2781 mg Ca kg^−1^; and 2083–2900 mg Mg kg^−1^. The levels of microelements were as follows: 2.1–22.0 mg Cu kg^−1^; 22.5–152.80 mg Fe kg^−1^; 31–109 mg Zn kg^−1^; and 17.53–2277.16 mg Mn kg^−1^ [22, 23], which is consistent with the values obtained in this study.

Among the analysed leguminous plants, the Andean lupine was characterised by a particularly high level of magnesium and potassium (*P* < 0.05) as well as copper, zinc, and iron (*P* < 0.01). The Andean lupine (*Lupinus mutabilis* Sweet), also called Chocho or Tarwi, originates from Latin America, where it is widely used as food and for medicinal purposes [24]. In turn, chickpea and lentil seeds can be a valuable element of diets, as they provide large amounts of calcium (6–5.5 g kg^−1^ DM). Similarly, the lentil seeds were shown to have substantial amounts of iron (63 mg kg^−1^ DM). The main factor that limits utilisation of mineral compounds from legume seeds is the presence of phytic acid, which reduces the bioavailability of minerals through chelation of such cations as zinc, copper, cobalt, iron, calcium, potassium, and magnesium and formation of non-absorbable phytates [25]. The content of phytic acid is below 1% in cultivated lupines, up to 1.6% in soybean, and up to 2% in bean seeds, i.e. these values are lower than those reported for barley or wheat [26]. Food processing methods, e.g. soaking, germination, fermentation, grinding, or cooking, effectively inactivate or reduce the impact of antinutritional components such as tannins, trypsin inhibitors, and phytic acid, which limit nutrient availability [27].

### Fatty Acid Profile of Legume Seeds

As shown in this study, the fatty acid profile in the fat of the legume seeds differs considerably between the Fabaceae species and even between their varieties (Table 3). The Andean lupine seeds were the richest in fat [13]; the amounts of fatty acids in the seed of Andean lupine were in the order of UFA > MUFA > PUFA > SFA. In turn, the lowest amounts of UFA, MUFA, and PUFA were found in the lentil, grass pea, broad bean, and pea seeds, compared with the content of these fatty acids in the seeds of lupines and chickpeas (respectively, *P* = 0.001, *P* = 0.001, and *P* = 0.005). The white and Andean lupines are similar to soybean in their fat content but different in terms of the percentage proportion of the individual fatty acid groups [28]. Soybean fat (199.4 g kg^−1^ of dry seeds) consists of ca. 14% of saturated fatty acids (primarily palmitic and stearic acids), 22% of monounsaturated fatty acids (oleic acid), and 56% of polyunsaturated fatty acids (linoleic and linolenic acids) [29, 30]. In the present study, the other legume seeds exhibited a similar profile of fatty acids to the profile determined in the soybean. In contrast to the other species (blue lupine, chickpea, pea, broad bean, yellow lupine, lentil, common bean, and grass pea), which contained from 38 to 17% MUFA and 41 to 64% PUFA of total fatty acids, the white and Andean lupines had a higher percentage of monounsaturated (*P* = 0.012) than polyunsaturated fatty acids (*P* = 0.017) (white lupine—66% MUFA and 22% PUFA; Andean lupine—44% MUFA and 36% PUFA). Similar results of the fatty acid profile in white lupine seeds (53.9% MUFA and 28.9% PUFA) were reported by Kalogeropoulos et al. [31]. Among the monounsaturated fatty acids, significant differences were found in the contribution of oleic acid. Its highest levels were determined in the white lupine fat (57.8 g in 100 g^−1^ fat) and the lowest content was noted in the grass pea and common bean (15.1 g in 100 g^−1^ fat) (*P* < 0.001). Similarly, large proportions of monounsaturated fatty acids, accounting for ca. 50% of total fatty acids, were determined for peanut fat; in this species, polyunsaturated fatty acids represent ca. 32% and saturated fatty acids account for 13% [30, 32]. Monounsaturated fatty acids can be utilised as an energy source. Moreover, they do not exert a negative effect on lipoproteins and blood clotting; in contrast, their positive effect has been noted, i.e. an increase in the proportion of HDL and a decrease in the level of LDL. Monounsaturated fatty acids are effective (in particular oleic acid) in lowering plasma cholesterol levels. Their recommended dietary intake is 15% of the energy demand, which is higher than the recommended PUFA (approximately 10% of the total energy can come from longer-chain *n*-3 or *n*-6 fatty acids) and SFA levels, with a recommended intake of 10% E [33].

**Table 3:** Fatty acid profiles in some leguminous species

Species		Fatty acids, g in 100 g^−1^ fat															Nutrition and dietetic value					Fatty acids, g in 100 g^−1^ seeds				
		C14	C16	C18	C20	C24	C16:1	C18:1 *n*-9	C18:1 *n*-7	C20:1 *n*-9	C22:1	C18:2 *n*-6	C18:3 *n*-3	Other FA	Medium chain (C14–C16)	Long chain (≥C18)	SFA:UFA	DFA	OFA	DFA: OFA	AI	C18:3 *n*-3	SFA	UFA	MUFA	PUFA
Blue lupine	cv. Regent	0.16	9.48	6.66	0.96	0.41	0.12	36.81	0.31	0.34	0.09	39.67	3.91	1.08	9.76	89.16	0.22	87.91	9.64	9.12	0.12	0.25	1.11	5.10	2.37	2.74
	cv. Zeus	0.21	12.63	4.79	2.61	0.56	0.19	36.25	0.63	0.33	0.24	33.69	4.72	3.15	13.03	83.82	0.27	80.84	12.84	6.30	0.18	0.20	0.89	3.27	1.62	1.65
	Mean value	0.19	11.06	5.73^a^	1.79	0.49	0.16	36.53^b^	0.47	0.34	0.17	36.68	4.32^b^	2.12	11.40	86.49	0.25	84.38	11.24	7.71	0.15	0.22^b^	1.00^b^	4.19^b^	1.99^b^	2.19^b^
White lupine	cv. Butan	0.09	6.54	1.59	1.08	0.64	0.09	58.11	1.75	5.46	0.74	14.23	7.58	2.10	6.72	91.18	0.11	89.55	6.63	13.51	0.08	0.73	0.95	8.42	6.33	2.09
	cv. Bardo	0.10	7.31	1.85	1.55	0.49	0.30	57.46	1.23	6.02	0.53	16.18	6.86	0.12	7.71	92.17	0.13	90.43	7.41	12.20	0.09	0.62	1.02	8.01	5.92	2.08
	Mean value	0.10	6.93	1.72^b^	1.32	0.57	0.20	57.79^a^	1.49	5.74	0.64	15.21	7.22^ab^	1.11	7.22	91.68	0.12	89.99	7.02	12.86	0.09	0.67^a^	0.99^b^	8.21^a^	6.13^a^	2.09^b^
Yellow lupine	cv. Parys	0.16	4.97	2.98	2.98	0.16	0.33	22.72	0.29	1.74	0.63	50.88	8.31	3.85	5.46	90.69	0.13	87.88	5.13	17.13	0.07	0.43	0.58	4.41	1.33	3.07
	cv. Iryd	0.26	9.81	2.75	2.77	0.18	0.46	22.51	0.18	2.01	0.59	47.49	7.65	3.34	10.53	86.13	0.19	83.64	10.07	8.31	0.13	0.28	0.58	2.95	0.94	2.01
	Mean value	0.21	7.39	2.87^b^	2.88	0.17	0.40	22.62^cd^	0.24	1.88	0.61	49.19	7.98^ab^	3.60	8.00	88.41	0.16	85.76	7.60	12.72	0.10	0.36^ab^	0.58^bc^	3.68^b^	1.14^b^	2.54^b^
Andean lupine		0.14	12.27	6.39^a^	0.74	0.12	0.31	43.06^b^	0.69	0.03	0.04	33.35	2.29^b^	0.57	12.72	86.71	0.25	86.16	12.41	6.94	0.16	0.33^ab^	2.81^a^	11.41^a^	6.31^a^	5.10^a^
Pea	cv. Medal	0.26	8.37	5.14	1.12	0.08	0.23	25.05	0.46	0.52	0.02	45.74	12.07	0.94	8.86	90.20	0.18	89.23	8.63	10.34	0.11	0.14	0.17	0.98	0.30	0.67
	cv. Mentor	0.49	10.08	4.36	0.97	0.11	0.15	32.16	0.47	0.68	0.09	41.49	8.76	0.19	10.72	89.09	0.19	88.16	10.57	8.34	0.14	0.08	0.14	0.74	0.30	0.44
	Mean value	0.38	9.23	4.75^a^	1.05	0.10	0.19	28.61^c^	0.47	0.60	0.06	43.62	10.42^a^	0.57	9.79	89.65	0.19	88.70	9.60	9.34	0.13	0.11^c^	0.16^c^	0.86^c^	0.30^c^	0.56^c^
Chickpeas		0.19	9.77	1.88^b^	0.78	0.02	0.09	32.36^b^	1.21	0.52	0.03	50.55	2.42^b^	0.18	10.05	89.77	0.14	89.06	9.96	8.94	0.12	0.12^c^	0.64^bc^	4.45^b^	1.74^b^	2.70^b^
Lentil		0.54	12.39	2.71^b^	1.51	0.52	0.21	20.05^cd^	0.82	1.11	0.16	44.74	13.79^a^	1.45	13.14	85.41	0.22	83.59	12.93	6.46	0.18	0.10^c^	0.13^c^	0.59^c^	0.16^c^	0.43^c^
Grass pea	cv. Derek	0.41	9.58	5.58	1.44	0.07	0.47	14.02	0.99	0.49	0.23	55.55	9.62	1.55	10.46	87.99	0.21	86.95	9.99	8.70	0.14	0.09	0.15	0.73	0.15	0.59
	cv. Krab	0.39	10.76	6.04	1.09	0.09	0.24	16.07	0.57	0.37	0.19	56.01	7.59	0.59	11.39	88.02	0.23	87.08	11.15	7.81	0.15	0.06	0.14	0.60	0.13	0.47
	Mean value	0.40	10.17	5.81^a^	1.27	0.08	0.36	15.05^d^	0.78	0.43	0.21	55.78	8.61^ab^	1.07	10.93	88.01	0.22	87.02	10.57	8.26	0.15	0.07^cd^	0.15^c^	0.67^d^	0.14^c^	0.53^c^
Common bean	cv. Mela	0.26	19.75	4.43^a^	0.97	0.87	0.21	15.07^d^	2.73	0.57	0.07	41.45	11.93^a^	1.69	20.22	78.09	0.36	76.46	20.01	3.82	0.29	0.26^b^	0.56^bc^	1.55^c^	0.40^c^	1.15^bc^
Broad bean	cv. Bonus	0.31	11.71	3.06^ab^	1.63	0.11	0.32	24.08^cd^	0.79	1.13	0.37	51.96	3.79^b^	0.74	12.34	86.92	0.20	85.50	12.02	7.11	0.16	0.03^d^	0.14^c^	0.70^d^	0.23^c^	0.47^c^
Pooled SEM		0.036	0.879	0.446	0.189	0.068	0.030	3.618	0.171	0.474	0.063	3.286	0.912	0.309	0.889	0.906	0.016	0.963	0.890	0.843	0.014	0.053	0.179	0.878	0.603	0.343
*P* value		0.071	0.066	0.011	0.214	0.31	0.452	<0.001	0.128	0.167	0.131	0.176	0.028	0.293	0.075	0.109	0.172	0.158	0.070	0.426	0.051	0.003	0.002	0.001	0.002	0.005

Values are mean of triplicate analyses

*DFA* desirable fatty acids DFA = (UFA + C18:0), *OFA* undesirable hypercholesterolemic fatty acids OFA = (C14:0 + C16:0), *AI* atherogenicity index AI = (4 × C14:0 + C16:0)/(UFA)

^abcd^Statistical differences between mean values (*P* < 0.05)

The fat in Fabaceae species seeds mainly contains exogenous fatty acids, i.e. linoleic acid (LA, 18:2, *n*-6) (21–53%) and α-linolenic acid (ALA, 18:3, *n*-3) (2.5–22%) [2, 8, 34]. This has also been confirmed in the present study showing their highest content, i.e. over 55% of total fatty acids, in the grass pea, lentil, yellow lupine, and broad bean seeds. ALA (*n*-3), which is a precursor of eicosapentaenoic acid (EPA) and docosahexaenoic acid (DHA), is insufficiently supplied in the traditional western diet. However, due to the low content of fat in legume seeds and the low levels of consumption thereof, the contribution of the seeds in increasing the daily supply of PUFA, in particular α-linolenic acid, in the diet is limited [34]. The recommended dietary allowance for α-linolenic acid is 1.1–1.6 g day^−1^, depending on the sex and physiological condition [33]. The highest contribution of ALA was noted in the lentil (13.8 in 100 g^−1^ fat), common bean (11.9 in 100 g^−1^ fat), and pea seed fat (10.4 in 100 g^−1^ fat) and the lowest levels were determined in the chickpea, Andean lupine, broad bean, and blue lupine seeds (*P* < 0.05) (Table 3). In terms of its content per 100 g in legume seeds, the white lupine (0.67 g), yellow lupine (0.36 g), and Andean lupine (0.33 g) seem to be the richest sources of this fatty acid, whereas the broad bean seeds are the poorest source (0.03 g). The level of ALA in the legume seeds mentioned above accounts for 42, 22.5, 21, and 2%, respectively, of the recommended daily amount [33]. Hence, these seeds can only partially cover the demand for this component in the diet. As reported by Kalogeropoulos et al. [31], the fat of legumes was rich in ALA, which represented 2.5–41.7% of all fatty acids. The authors determined the percent coverage of daily supply for ALA by consuming one serving (125 g) of cooked dry legumes. They obtained very similar coverage of daily supply of this fatty acid, i.e. 35.6% for the white lupine and 4% for the broad bean, as indicated by the results presented in this paper.

Among saturated fatty acids, the contribution of stearic acid (in 100 g^−1^ fat) in the white lupine, chickpea, lentil, yellow lupine, and broad bean seeds was lower than that in the Andean lupine, grass pea, blue lupine, pea, and common bean seeds (*P* < 0.05) (Table 3). The most favourable DFA:OFA ratio was found for the lupine fat: yellow lupine—Paris cv. (17.1) and white lupine—Butan (13.5) as well as Bardo cvs. (12.2). The lowest AI was also characteristic for these seeds, whereas the highest AI value was noted in the seeds of the Mela cv. common bean. The analysed seeds were characterised by a very high proportion of DFA with a neutral effect on the cholesterol level or hypocholesterolemic activity (on average 86%) and simultaneously with a low atherogenicity index (on average 0.15). In vivo animal and human investigations indicate great cardioprotective potential of legume seed fat [5, 35].

### Correlation Between Nutrients and Phytochemical Compounds and Antioxidant Activities

Legume seeds with their high contents of nutrients and bioactive components exhibit significant antioxidant activity [13, 36, 37]. Statistical relationships between the 2,2-diphenyl-1-picrylhydrazyl (DPPH) antioxidant activity [13] and the proportion of fatty acids as well as the content of phenols and tannins [13] and the mineral composition of the analysed legume seeds were determined (Table 4).

**Table 4:** Correlation coefficient between the antioxidant activity and phytochemical contents and some nutrients in legume seeds

Variables	Blue lupine	White lupine	Yellow lupine	Andean lupine	Pea	Chickpea	Lentil	Grass pea	Common bean	Broad bean
DPPH										
SFA	0.193	0.097	0.278	0.358	−0.356	0.123	0.489	0.409	0.178	0.245
UFA	0.687*	0.647*	0.876*	0.647*	0.707*	0.598*	0.612*	0.579*	0.642*	0.734*
MUFA	0.219	−0.186	0.547	0.474	−0.168	0.273	0.322	0.468	0.367	0.473
PUFA	0.693*	0.645*	0.837*	0.745*	0.691*	0.583*	0.764*	0.581*	0.607*	0.468
Total phenols										
K	0.478	0.597*	−0.114	0.207	0.187	0.211	0.213	0.451*	0.197	0.243
Na	−0.398*	−0.246	0.143	0.513*	0.481*	0.289	0.227	0.549*	0.319	0.349
P	−0.246	−0.448*	0.463*	0.319	0.532*	0.427	0.576*	0.439*	0.308	0.434*
Ca	−0.344	−0.401*	0.507*	0.546*	0.240	0.273	0.242	0.341	0.287	0.423*
Mg	0.439*	0.517*	0.546*	0.498*	0.516*	0.456*	0.529*	0.436*	0.291	0.464*
Mn	0.364	0.238	0.179	0.087	0.127	0.123	0.238	0.141	0.278	0.237
Fe	−0.128	0.243	0.518*	0.216	0.343	0.298	0.249	−0.119	0.243	0.278
Zn	0.094	0.216	0.152	0.178	0.378	0.242	0.291	0.159	0.249	0.291
Cu	0.318	−0.224	0.427*	0.298	0.434*	0.256	0.394	0.179	0.127	0.519*
Tannins										
K	−0.619*	0.673*	0.574*	0.489	0.513	0.421	0.348	0.743*	0.678*	0.435
Na	−0.102	−0.097	−0.258	0.343	−0.264	0.071	0.217	0.319	0.348	0.419
P	−0.535*	−0.517*	−0.627*	−0.541*	0.457	0.499	0.434	0.568*	0.561*	0.606*
Ca	−0.473	−0.548	−0.493	0.234	0.445	0.313	0.326	0.459	0.347	0.578*
Mg	−0.234	−0.174	−0.467*	0.273	0.387	0.246	0.463*	0.504*	0.237	0.298
Mn	−0.563*	−0.388	0.458*	0.207	−0.446*	0.352	0.287	0.139	0.498*	0.349
Fe	0.109	0.099	−0.216	0.342	0.114	0.187	0.292	−0.351	0.187	0.248
Zn	0.515*	0.523*	0.475*	0.465*	0.447*	0.501*	0.378	0.284	0.491	0.339
Cu	0.179	0.187	0.276	0.187	0.545*	0.217	0.298	0.393	0.238	0.342

*Statistical differences (*P* < 0.05)

Factors that have an effect on the rate of oxidative changes in vegetable fats include the chemical structure of fatty acids in the triacylglycerol molecule, the number and site of unsaturated bonds, and the presence of pro-oxidant or antioxidant compounds [9, 10, 38]. The UFA contribution is a very important determinant of fat stability, which is associated with chemical reactions occurring on double bonds. The oxidation rate depends on the number of double bonds in the carbon chain. Therefore, the susceptibility to oxidation increases exponentially in proportion with the number of unsaturated bonds in the fatty acids. Fats with a high proportion of oleic acid will be less susceptible to *n*-6 PUFA oxidation (linoleic acid) [39, 40]. In this study, the antioxidant activity determined with the DPPH assay proved to be highly positively correlated with the content of UFA (0.5 ≤ *r* < 1; *P* < 0.05) in all seeds; in turn, such a correlation in the case of PUFA was shown for the lupine, pea, chickpea, lentil, grass pea, and common bean seeds (Table 4). Some studies indicate an ability of long-chain polyunsaturated fatty acids to act as antioxidants. Reduced excretion of lipid peroxidation products, lower production of reactive oxygen species (ROS), and direct superoxide scavenging by LC-PUFA, especially by those from the *n*-3 group, have been shown in in vivo data [35]. This was confirmed by evaluation of the interactions between the degree of DPPH free radical scavenging and the content of polyunsaturated fatty acids in quinoa seeds (*Chenopodium quinoa* Willd.) and avocado (*Persea americana* Mill.) [41, 42]. These correlations were similar to those noted in the present study. In contrast, no correlation between the UFA, MUFA, and PUFA content and DPPH antioxidant activity was reported in investigations carried out by Zhang et al. [12]. According to the authors, this suggests that the content of the individual groups of fatty acids was not related to the antioxidant activity. The significant positive correlation between the antioxidant DPPH activity and the UFA and PUFA proportions noted in this study suggests that other chemical components can exert a significant effect on the oxidative stability of fatty acids. For instance, ascorbic acid, tocopherols, and phenolic compounds can partly contribute to the increase in the antioxidant activity [43, 44]. There are also reports presenting higher efficiency of water-soluble compounds, such as phenolic compounds, in protecting fatty acids against oxidation, than ascorbate or lipid-soluble compounds, e.g. tocopherol and lutein [45, 46]. This correlation has been confirmed by other authors, who reported plant capability of accumulation of antioxidant components in order to enhance the oxidative stability of polyunsaturated fatty acids [47–50].

In the analysed seeds, the strength and direction of the correlation between the content of mineral elements and the total content of phenols and tannins were assessed (Table 4). Polyphenols are natural antioxidants scavenging free radicals, binding transition metal ions (Fe^2+^ and Cu^2+^), and preventing lipid peroxidation. Besides phenolic compounds, antioxidant activity is attributed to mineral components such as copper, manganese, and iron [8–10]. The total phenols were highly correlated (0.5 ≤ *r* < 1) with the content of K (white lupine [+]), Na (Andean lupine [−] and grass pea [+]), P (pea and lentil [+]), Ca (yellow and Andean lupines [+]), Mg (white and yellow lupines, pea, and lentil [+]), Fe (yellow lupine [+]), and Cu (broad bean [+]) (*P* < 0.05). In the other cases, these relationships were correlated poorly or moderately. Polyphenols are related to the ability to bind to transitional metal ions [51]. These compounds have been found to concentrate primarily in the legume seed coats, and seeds of plants producing coloured flowers contain greater levels thereof [52–55]. In the study by Troszyńska et al. [54], it was found that the coloured seed coats of faba bean, broad bean, and pea and lentil seeds were the richest in phenolic compounds, in which flavanols represented from 55.0 to 78.3% of the total phenolics. In the present study, this has been confirmed by the correlation between the total phenols and the content of elements in the white bean seed, where no significant relationships were found between these variables. In turn, in the case of all the other analysed legume seeds with a coloured seed coat (Table 1), there were significant correlations between the total phenolic compounds and the different elements. Similarly, Cairns et al. [56] found that the organometallic forms of Cd, Cu, Hg, Mn, Mo, Ni, Pb, Sr, and Zn in tea were primarily associated with the flavanoid components. In investigations of medicinal plants [57], significant relations between metallic elements (Zn, Mn, and Cu) and *p*-coumaric acid were reported. The results corroborate the values determined for the total phenol-Cu pair in the yellow lupine, pea, and broad bean seeds (*r* = 0.427, *r* = 0.434, and *r* = 0.519, respectively; *P* < 0.05) (Table 4).

Leguminous plants contain compounds, e.g. tannins, which diminish their nutritional value by reduction of the digestibility and bioavailability of nutrients, including minerals [6, 25]. Tannins are phenol compounds present in a majority of legume seeds and found mainly in coloured seed coats. Their physiological functions consist in plant protection against the detrimental action of insects and microorganisms [49]. Tannins can interact with elements, e.g. iron, calcium [11], zinc [58], and sodium [59], mainly by chelation and inhibition of digestive enzymes, which ultimately results in reduced availability of minerals for absorption in the gastrointestinal tract [11]. Different relationships have been observed in the present investigations. A high correlation (0.5 ≤ *r* < 1) was determined between tannins and K (blue lupine [−], white and yellow lupines, grass pea, and common bean [+]), P (lupines [−], grass pea, common bean, and broad bean [+]), Ca (broad bean [+]), Mg (grass pea [+]), Mn (blue lupine [−]), and Cu (pea [+]) (*P* < 0.05). In turn, there were no significant correlations between the tannin content and Fe in the analysed seeds. Similarly, Santos et al. [60] and Welch et al. [61] did not observe a correlation between tannins and iron in legume seeds. These differences in the results may be associated with the fact that the content of hydrolysable tannins (pyrogallol-type tannins) was determined in the analysis presented in our previous studies [13]. The group of compounds referred to as tannins is highly chemically heterogeneous and is mainly divided into condensed and hydrolysable tannins. Condensed tannins are polymers of catechins or leucenanthoey anilins linked via acid-labile carbon-carbon bonds. Hydrolysable tannins are composed of gallic acid or its condensation product ellagic acid esterified to the hydroxyl groups of glucose [6]. Condensed tannins can form insoluble complexes with Fe [55, 62, 63]. In the analysed seed, moderate and strong positive correlations between the tannin content and Zn were noted in the lupines, pea, and chickpea (*P* < 0.05). Karamać [64] observed that the tannin fraction from walnuts contained mainly hydrolysable tannins hardly complexing Zn(II). Similarly, Santos et al. [60] found significant relationships between tannins and Zn, Mg, and Mn in common bean. In the present study, a moderate positive correlation was found between the tannin content and manganese in the common bean seeds and between tannins and magnesium in the lentil and grass pea seeds (*P* < 0.05). The correlations between the phenolic compounds and elements may be associated with the chemical structure and the probable role of these elements in the biosynthesis of the phenolic compounds.

## Conclusions

In summary, the analysed Fabaceae seeds were characterised by a highly diverse mineral composition and fatty acid profile, which was reflected in the multidirectional interactions between these nutrients and phytochemicals and antioxidant activity. The positive correlation between the unsaturated fatty acids and antioxidant activity, the predominant proportion of hypocholesterolemic fatty acids, and the low atherogenicity index of the fatty acids contained in legume plant seeds indicate their great protective potential in the prevention and treatment of diet-dependent diseases. Given these characteristics, legume seeds can again become a valuable part of the diet.
